# Prospective pilot study of tirofiban in progressive stroke after intravenous thrombolysis

**DOI:** 10.3389/fneur.2022.982684

**Published:** 2022-10-04

**Authors:** Yan Zhang, Jianliang Wang, Zhaoxi Ma, Guihua Mu, Da Liang, Yifan Li, Xiaoyan Qian, Luyuan Zhang, Fang Shen, Lei Zhang, Jie Yu, Yang Liu

**Affiliations:** ^1^Department of Neurology, The Kunshan Affiliated Hospital of Jiangsu University, The First People's Hospital of Kunshan, Kunshan, China; ^2^Department of Radiology, The Kunshan Affiliated Hospital of Jiangsu University, The First People's Hospital of Kunshan, Kunshan, China; ^3^Department of Scientific and Technological Talents, The Kunshan Affiliated Hospital of Jiangsu University, The First People's Hospital of Kunshan, Kunshan, China; ^4^Department of Outpatient, The Kunshan Affiliated Hospital of Jiangsu University, The First People's Hospital of Kunshan, Kunshan, China; ^5^Department of Neurology, The Second People's Hospital of Kunshan, Kunshan, China; ^6^Department of Neurology, Saarland University, Homburg, Germany

**Keywords:** cerebrovascular disease, stroke, thrombolytic therapy, antiplatelet therapy, tirofiban

## Abstract

**Background:**

Intravenous thrombolysis (IVT) is a standard procedure for the treatment of patients with acute ischemic stroke (AIS). Improving the therapeutic efficacy of IVT is an important task for neurologists. The aim of this study was to evaluate the efficacy and safety of early low-dose tirofiban treatment in AIS patients with early neurological deterioration (END) after IVT.

**Methods:**

In this prospective and randomized pilot study, 73 AIS patients with END were recruited from a local hospital in China. Of these, 14 patients were treated with regular antiplatelet agents (aspirin plus clopidogrel) and 59 patients were treated with tirofiban within 24 h of IVT, followed by regular antiplatelet therapy. Neurological deficits and functional recovery were assessed with NIHSS and modified Rankin Scale (mRS) at 7 and 90 days. During the 90-day follow-up period, both hemorrhagic (e.g., intracerebral hemorrhage) and non-hemorrhagic (e.g., pneumonia) events were recorded.

**Results:**

Treatment with tirofiban compared with regular antiplatelet therapy: (1) improved functional recovery of AIS patients to mRS (≤2) at both 7 and 90 days (odds ratios [ORs], 1.37 and 1.64; 95% confidence interval [CI], 1.16–1.61 and 1.26–2.12; *P* = 0.008 and < 0.001, respectively), and (2) reduced NIHSS scores from 11.14 ± 2.38 to 5.95 ± 3.48 at day 7 (*P* < 0.001) and from 8.14 ± 2.74 to 4.08 ± 3.50 at day 90 (*P* < 0.001). Tirofiban treatment did not increase the risk of hemorrhagic complications. Multivariate regression analysis showed that tirofiban treatment independently predicted a favorable functional outcome (*P* ≤ 0.001).

**Conclusion:**

Early treatment with low-dose tirofiban in AIS patients with neurologic deterioration after IVT potentially improved functional recovery and attenuated neurologic deficits as early as 7 days and did not increase the risk of various hemorrhagic complications. However, the therapeutic efficacy of tirofiban treatment in END patients needs to be determined by future randomized clinical trials with a large study population.

**Clinical trial registration:**

http://www.chictr.org.cn/, Identifier ChiCTR2200058513.

## Background

Acute ischemic stroke (AIS) is a leading cause of disability and death in China and worldwide (1, 2). Intravenous thrombolysis or/and intraarterial thrombectomy to reopen occluded cerebral arteries is a standard treatment procedure for AIS patients. There is evidence that thrombectomy has a higher recanalization rate than thrombolysis in large vessels; however, endovascular therapy can only be performed in selected high-performing stroke centers. In smaller hospitals, especially in rural areas, intravenous thrombolysis with recombinant tissue-type plasminogen activator (rt-PA) remains the mainstay for AIS patients (3, 4). Further studies on how to improve the therapeutic efficacy of intravenous thrombolysis are always important.

Early neurological deterioration (END) within the first 24 h after intravenous thrombolysis is a major problem leading to poor outcomes in AIS patients (5). Using the definition of a National Institutes of Health Stroke Scale (NIHSS) change ≥4, the incidence of END after thrombolysis is approximately 14% (6). The pathogenic mechanisms mediating END are largely unknown, and specific medical treatments that prevent or reverse END are lacking. Recently, it has been suggested that extension of the initial thrombus or new embolic events in the same ischemic brain region may further impair brain perfusion and cause END in AIS patients. Therefore, antiplatelet therapy that stops thrombus formation may be helpful to prevent END (5).

Tirofiban is a non-peptide antagonist of the major platelet surface receptor glycoprotein IIb/IIIa. It prevents fibrinogen from binding to glycoprotein IIb/IIIa, thus blocking platelet aggregation. Its half-life in plasma is 1.5–2 h. After discontinuation of tirofiban administration, platelet aggregation recovers to 50% of baseline within 4 h (7). Interestingly, a recent multicenter retrospective study showed that the use of tirofiban at a low dose in patients with END within the first 24 h after intravenous thrombolysis improves functional outcome at 3 months, as assessed by the modified Rankin Scale (mRS), and does not increase the risk of intracerebral hemorrhage (8). In another study, early administration of tirofiban after intravenous thrombolysis (within 2 or 12 h) showed better efficacy than late administration (after 12 h) in reducing NIHSS and mRS within 2 weeks and at 3 months (9).

In our prospective pilot study, we selected AIS patients with END with an NIHSS increase ≥4 within 24 h after intravenous thrombolysis as the study subjects and treated them with a low dose of tirofiban for up to 24 h followed by regular antithrombotic drugs (e.g., aspirin and clopidogrel). The aim of our study was to investigate whether treatment with tirofiban prevents further neurological deterioration and improves functional recovery in AIS patients with END.

## Methods

### Study design and participants

Our study was an open-label, randomized and prospective trial and conducted according to the principles of the Declaration of Helsinki. The trial protocol was approved by the Ethics Committee of the Kunshan Affiliated Hospital of Jiangsu University. All participants or their legal representatives provided written consent for data collection. Our trial was retrospectively registered on the Chinese Clinical Trial Registry (http://www.chictr.org.cn; ChiCTR2200058513). The calculation of sample size was not performed because no comparisons had shown the significant therapeutic efficacy of tirofiban in thrombolysis-treated AIS patients with END before the beginning of our study. However, we recruited at least 12 patients for each group, as increasing the sample size to 12 participants greatly improves the precision of the mean and variance of a research population, whereas increasing the sample size beyond 12 participants does not (10).

Between June 2018 and May 2022, 378 AIS patients aged ≥18 years at the First People's Hospital of Kunshan, China, received intravenous thrombolysis with rt-PA, alteplase (0.9 mg/kg) within 4.5 h of stroke onset according to the international guidelines (11, 12) and the guideline prepared by Chinese Stroke Association Stroke Council Guideline Writing Committee (13). At 24 h after thrombolysis or at any time within 24 h neurological deficits progressed, patients were reexamined with head computed tomography (CT) and reassessed with the NIHSS. All of these patients with an increase in NIHSS ≥4 (including patients in whom NIHSS scores initially decreased and then increased) were diagnosed as END (5).

The inclusion criteria for this study were as follows: AIS patients with END after intravenous thrombolysis with rt-PA as described above. The exclusion criteria were: the head CT scan showing intracerebral hemorrhage and malignant edema; all bleeding disorders outside the brain, which required medical intervention; known thrombocytopenia or a thrombocyte count ≤100 × 10^9^/L; severe cardiac, hepatic or renal insufficiency; intolerance to intravenous infusion of tirofiban; and missing written informed consent. Because there was no clear evidence of platelet activation in cardioembolic stroke patients (14), AIS patients with suspected cardiogenic causes (e.g., atrial fibrillation) were also excluded from this study.

### Randomization and treatments

Eligible patients were randomly assigned (4:1) to receive tirofiban or regular treatment alone (control), i.e., after 4 END patients were treated with tirofiban, the next (fifth) received regular antiplatelet therapy. Dr. Yan Zhang coordinated the entire study. All participating physicians and patients were aware of the treatment protocols. In the control group, regular treatment consisted of aspirin at a dose of 100 mg plus clopidogrel at a dose of 75 mg per day, starting 24 h after intravenous thrombolysis, followed by different therapies according to the classification of stroke subtypes (15): (1) continuation of aspirin plus clopidogrel in patients with large artery atherosclerosis; and (2) replacement with aspirin alone starting 3 weeks after intravenous thrombolysis in patients with small vessel occlusion. In the tirofiban group, patients received tirofiban hydrochloride (Grand Pharmaceutical Co Ltd, Wuhan, China) infusion, a loading dose of 0.4 μg/kg/minute over 30 min followed by a maintenance dose of 0.1 μg/kg/minute up to 24 h, starting immediately after END was diagnosed. Subsequently, regular treatment was initiated 4 h before the completion of tirofiban infusion. Other strategies of treatment included statins, management of blood glucose, blood pressure, or combinations of these treatments, following the Chinese guidelines for the early management of AIS patients (13).

### Data collection

Baseline demographic and clinical information for all enrolled patients included age, sex, and presence of hypertension (previous use of antihypertensive agents), diabetes mellitus (previous use of hypoglycemics or serum random glucose level ≥11.1 mmol/L, or glycosylated hemoglobin >6.4% on admission), dyslipidemia (previous use of antihyperlipidemic agents or serum cholesterol level >5.17 mmol/L on admission), coronary artery disease, atrial fibrillation, current smoking (any cigarette usage within the 28 days preceding admission), previous stroke, previous use of antiplatelet drugs and anticoagulants, admission blood pressure, NIHSS scores at admission and after intravenous thrombolysis. Laboratory findings included glucose, cholesterol (TC) triglyceride (TG), high-density lipoprotein (HDL), low-density lipoprotein (LDL), homocysteine and fibrinogen levels in the serum. The number of white blood cells (WBC) and platelets was counted in the blood. Stroke subtypes were classified as large artery atherosclerosis, small vessel occlusion, cardioembolic, or undetermined cause according to the Trial of ORG 10172 in the Acute Stroke Treatment classification (TOAST) (15). Time from stroke onset to intravenous thrombolysis was also recorded. The time of stroke onset was defined as the time when the patient was last seen normal, and the time of intravenous thrombolysis was defined as the time at which alteplase was intravenously injected.

The primary efficacy outcome was the recovery of functional outcome as shown in mRS score (ranging from 0 [no symptoms] to 6 [death]) 7 days after intravenous thrombolysis (or at the hospital discharge) and at 90 days. The secondary efficacy outcome was the attenuation of neurological deficits as assessed by NIHSS scores (ranging from 0 [no stroke symptoms] to 42 [severe stroke]) at 7 days (or at the hospital discharge) and 90 days.

The primary safety outcome was both symptomatic and non-symptomatic intracranial hemorrhage as reflected by CT scanning or magnetic resonance imaging (MRI) within 7 days after intravenous thrombolysis. The secondary safety outcome was systemic bleeding requiring immediate medical intervention. Other safety outcomes included all-cause mortality and non-hemorrhagic serious adverse events (e.g., pneumonia, urinary tract infections and venous thrombosis) within 7 days after treatment.

### Statistical analysis

Calculations were performed using SPSS software for Windows (version 27.0, IBM, Armonk, USA). The data for continuous variables were described as mean ± SD, and categorical variables were presented as frequencies. Continuous variables were compared between tirofiban and control groups by the Mann-Whitney *U* test. Categorical variables were compared by Pearson χ^2^ test. To assess the efficacy of tirofiban infusion on the outcome of AIS patients, mRS at day 7 and 90 was analyzed as a categorical or ordinal variable. Odds ratios (ORs) with 95% confidence intervals were calculated for mRS ≤2 as a categorical variable representing favorable functional outcome. mRS as an ordinal variable was used as the dependent variable in ordinal logistic regression analysis. Total 4 models were created, in which tirofiban treatment, in combination with various pre-stroke risk factors, clinical and laboratory findings on admission, or complications during the 90-day follow-up period were used as independent variables. *P* < 0.05 was considered statistically significant. All reported *P* were 2-sided.

## Results

### Patient characteristics

**Figure 1:**
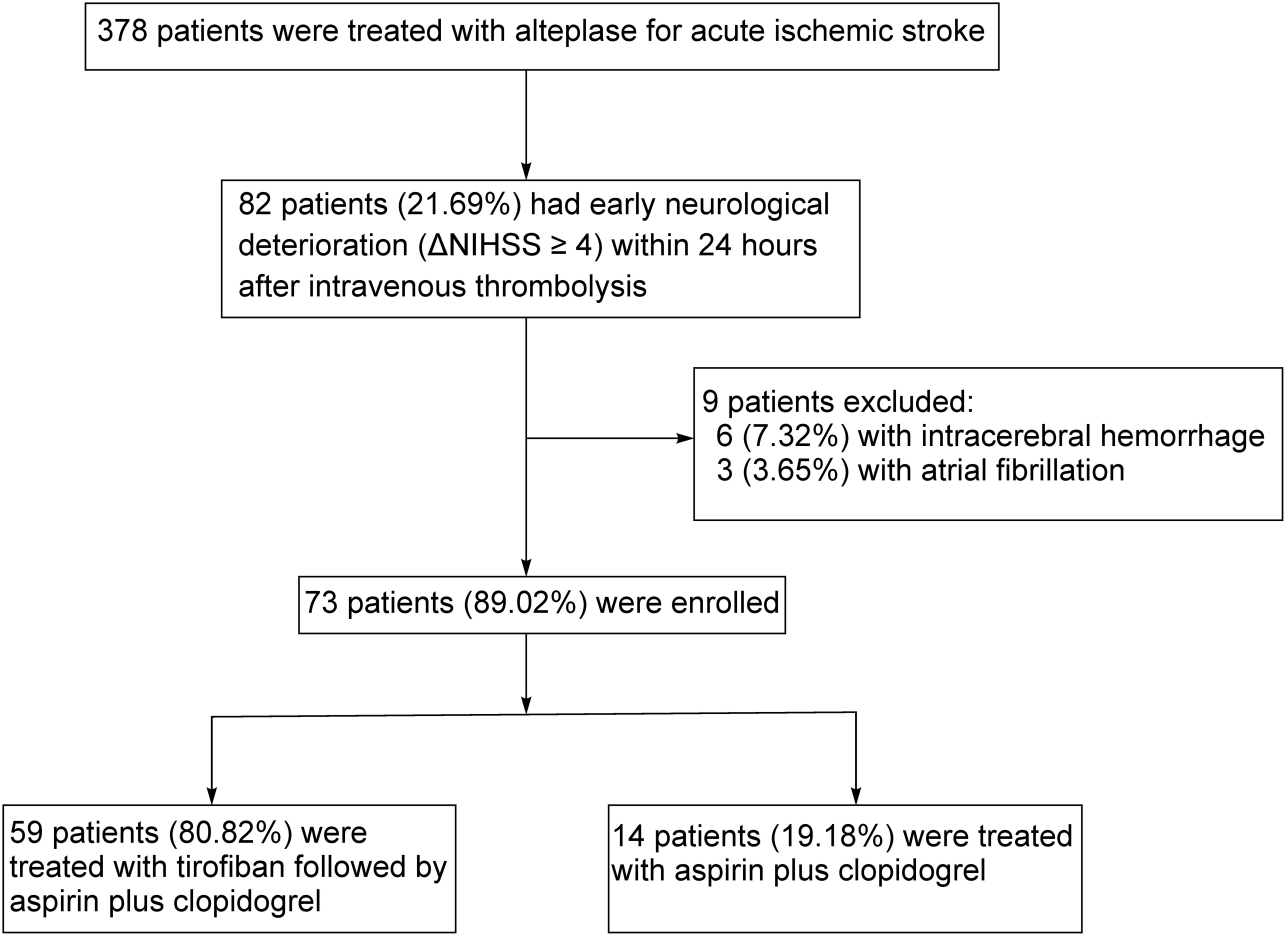
Study flow chart.

From June 2018 to May 2022, 378 AIS patients in our hospital received intravenous thrombolysis with rt-PA according to the international and Chinese guidelines (11–13). Eighty-two patients (21.69% of all AIS patients) had progressive neurological deficits within 24 h after therapy, as evidenced by an increase in NIHSS ≥4. These patients were defined as END patients. Six patients (7.32% of END patients) had intracerebral hemorrhage, as demonstrated by a CT scan of the head. No patient had malignant edema. Three END patients suffered from atrial fibrillation and a cardioembolic cause was suspected for the stroke. Finally, 73 END patients were enrolled and randomly assigned to tirofiban plus regular treatment with aspirin and clopidogrel (tirofiban group; *n* = 59) and regular treatment alone (control group; *n* = 14) (see Figure 1). No patient was lost during the 90-day follow-up period. There were no other END patients excluded from the study due to the contraindications to tirofiban treatment, including coagulopathy, anticoagulant therapy, thrombocytopenia, and hepatic or renal dysfunction.

The demographic and baseline characteristics of the cohort and of tirofiban and control groups of patients were shown in Table 1. The coronary artery disease was more frequent in tirofiban group (*P* < 0.05). All other analyzed parameters, including age, gender, pre-stroke risk factors (e.g., hypertension, diabetes mellitus, dyslipidemia, previous stroke and current smoking), clinical and laboratory findings at admission (e.g., blood pressure, admission NIHSS score, serum glucose and fibrinogen, et al.) and TOAST classification were not significantly different between tirofiban and control groups. Of note, there were no significant differences between the tirofiban and control groups in the percentage with pre-stroke mRS score of 0 [91.53 vs. 85.71%; χ^2^_(2)_ = 0.441, *P* = 0.507], in the time from stroke onset to intravenous thrombolysis (196.10 ± 53.89 vs. 209.00 ± 55.17 minutes; *U* = 352,000, *Z* = −0.855, *P* = 0.393), and in the increase of NIHSS scores within 24 h after thrombolysis (5.93 ± 1.24 vs. 5.29 ± 1.54; *U* = 293500, *Z* = −1.715, *P* = 0.086). Thus, these 2 comparable groups of END patients were suitable for analysis of the efficacy and safety of tirofiban treatments.

**Table 1:** Demographic and Baseline Characteristics of END patients.

**Variable**	**All patients**	**Tirofiban**	**No-tirofiban**	***P*-value**
	**(*n =* 73)**	**(*n =* 59)**	**(*n =* 14)**	
Age, year	69.04 ± 14.43	69.24 ± 14.88	68.21 ± 12.86	0.584
Women	46.58%	45.76%	50.00%	0.775
**Prestroke vascular risk factors**
Hypertension	76.71%	76.27%	78.57%	0.855
Diabetes mellitus	19.18%	16.95%	28.57%	0.321
Dyslipidemia	23.29%	23.73%	21.43%	0.855
Previous stroke	15.07%	11.86%	28.57%	0.116
Coronary artery disease	21.92/%	27.12%	0%	0.027
Current smoking	42.47%	47.46%	21.43%	0.077
**Clinical findings at admission**
SBP at baseline, mmHg	160.70 ± 17.38	161.20 ± 15.51	158.57 ± 24.25	0.153
DBP at baseline, mmHg	92.79 ± 15.95	92.58 ± 12.69	93.71 ± 26.23	0.828
Pre-stroke antiplatelet use	21.92%	23.73%	14.27%	0.443
Admission NIHSS score	8.75 ± 2.90	8.90 ± 2.75	8.14 ± 3.51	0.390
Increase of NIHSS score from admission after IVT	5.81 ± 1.32	5.93 ± 1.24	5.29 ± 1.54	0.086
Premorbid mRS score = 0	90.41%	91.53%	85.71%	0.507
Stroke onset to door, minutes	142.19 ± 52.94	142.54 ± 52.99	140.71 ± 54.70	0.915
Stroke onset to IVT, minutes	198.58 ± 53.99	196.10 ± 53.89	209.00 ± 55.17	0.393
**Laboratory findings at admission**
Glucose, mg/dL	6.01 ± 2.07	5.94 ± 2.20	6.27 ± 1.43	0.202
TC, mmol/L	4.51 ± 1.61	4.56 ± 1.68	4.26 ± 1.29	0.634
TG, mmol/L	1.52 ± 0.96	1.60 ± 1.03	1.17 ± 0.48	0.167
LDL, mmol/L	2.45 ± 1.07	2.44 ± 1.09	2.53 ± 1.09	0.604
HDL, mmol/L	1.27 ± 0.35	1.27 ± 0.37	1.28 ± 0.25	0.700
Homocysteine, μmol/L	17.06 ± 10.72	17.04 ± 9.61	17.13 ± 14.94	0.098
WBC, × 10^9^/L	7.06 ± 2.35	7.13 ± 2.52	6.77 ± 1.52	0.861
Platelet, × 10^9^/L	184.98 ± 52.05	187.93 ± 46.39	172.53 ± 72.08	0.806
Fibrinogen, g/L	3.14 ± 0.86	3.16 ± 0.84	3.07 ± 0.95	0.594
**TOAST classification**				0.080
Large artery atherosclerosis	65.75%	61.02%	85.71%	
Small vessel occlusion	34.25%	38.98%	14.29%	

Values are mean ± SD, or frequency (%). DBP indicates diastolic blood pressure; HDL, high-density lipoprotein; IVT, intravenous thrombolysis; LDL, low-density lipoprotein; NIHSS, National Institutes of Health Stroke Scale; SBP, systolic blood pressure; TC, total cholesterol; TG, triglyceride; TOAST, Trial of ORG 10172 in Acute Stroke Treatment; and WBC, white blood cell.

### Efficacy outcome

**Figure 2:**
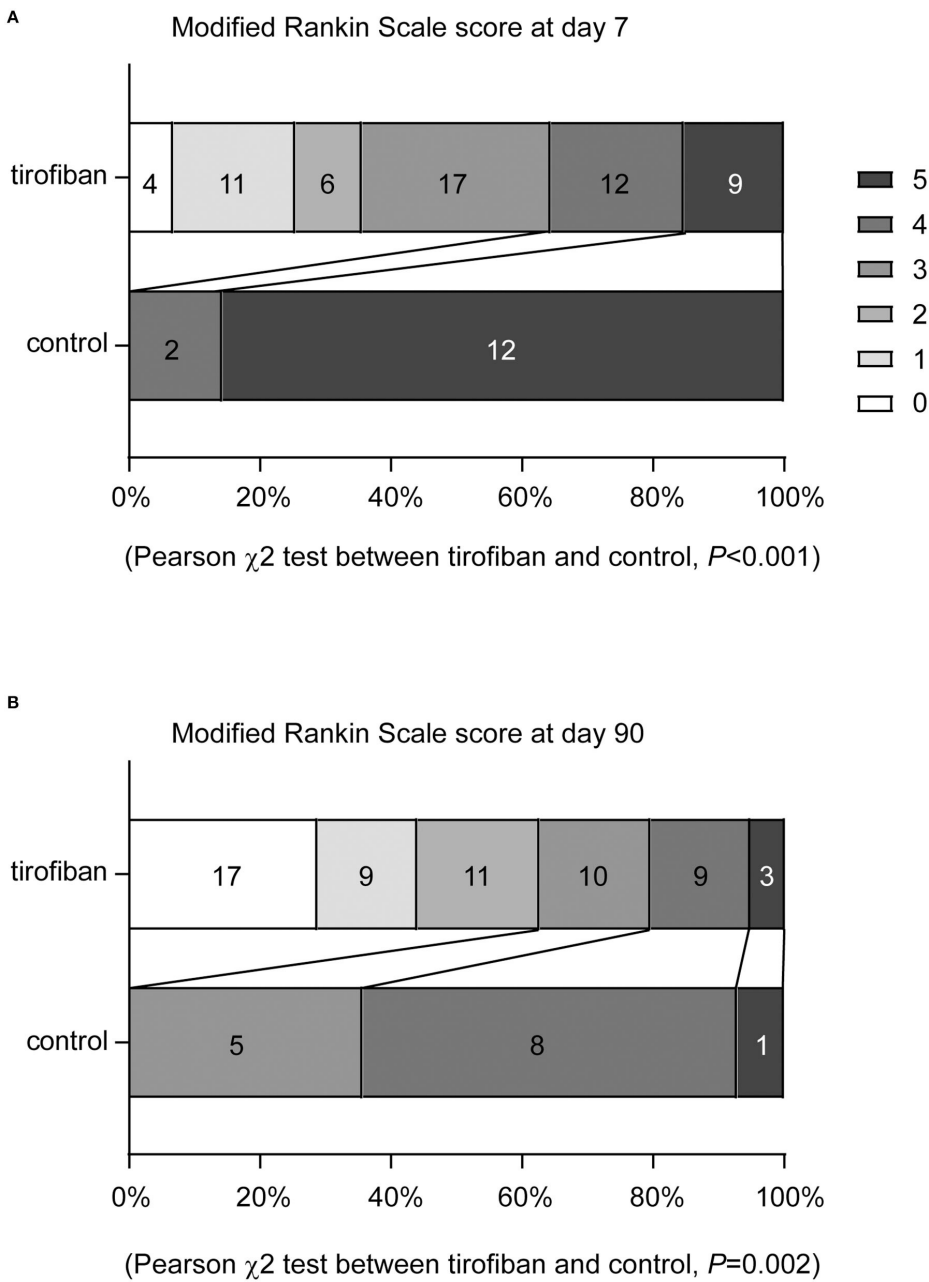
Tirofiban treatment shifts the distribution of modified Rankin Scale (mRS) scores toward to lower values at 7 and 90 days. The functional recovery of AIS patients were assessed with mRS at both 7 and 90 days. mRS scores range from 0 to 5 and score 0 indicates no symptoms, score 1 indicates no clinically significant disability, score 2 indicates slight disability, score 3 indicates moderate disability, score 4 indicates moderately severe disability, and score 5 indicates severe disability. Tirofiban treatment shifts the distribution of mRS scores toward to lower values at both day 7 **(A)** and 90 **(B)** compared with the control groups without tirofiban treatment. χ^2^ test, *n* = 59 and 14 for tirofiban and control groups, respectively.

To evaluate the efficacy of tirofiban treatment, we assessed mRS scores of AIS patients with END 7 and 90 days after intravenous thrombolysis. In the control group of patients receiving regular antiplatelet treatment, all 14 subjects failed to achieve a favorable outcome (mRS ≤2) at both time points, whereas 35.59% and 62.71% of AIS patients treated with tirofiban plus regular antiplatelet therapy reached mRS ≤2 in 7 and 90 days, respectively. More specifically, the distribution of mRS scores at both 7 and 90 days shifted significantly toward lower values in the tirofiban group compared with the control group (Figures 2A,B, Pearson χ^2^ test of mRS distribution between tirofiban and control groups, $\chi_{\left(5\right)}^{2}$ = 28.761 and 19.332, *P* < 0.001 and = 0.002, at day 7 and 90, respectively).

When favorable and poor functional outcomes were defined as mRS ≤2 and >2, the odds ratios (ORs) of tirofiban treatment vs. regular treatment for improving recovery of AIS patients were 1.37 and 1.64 at 7 and 90 days [Table 2; χ^2^_(1)_ = 6.995 and 17.803, *P* = 0.008 and < 0.001], respectively. In a subgroup analysis with exclusion of subjects with previous stroke, we observed that treatment with tirofiban was still able to benefit AIS patients in the functional recovery both at day 7 and at day 90 (ORs, 1.313 and 1.526; 95% confidence intervals, 1.108–1.554 and 1.172–1.988; χ^2^_(1)_ = 5.678 and 13.568, *P* = 0.017 and < 0.001; respectively). As tirofiban infusion has been reported to benefit AIS patients with large artery atherosclerosis more than patients with small vessel occlusion (16), we performed a second subgroup analysis with exclusion of patients with small vessel occlusion according to TOAST classification (15). Tirofiban treatment appeared to increase ORs of tirofiban treatment in improving functional recovery at day 7 and day 90 compared with ORs of tirofiban treatment in END patients with occlusion in large or small vessels [ORs, 1.545 and 1.923; 95% confidence intervals, 1.206–1.981 and 1.320–2.803; χ^2^_(1)_ = 6.588 and 14.720, *P* = 0.010 and <0.001; respectively].

**Table 2:** Functional recovery of END patients after intravenous thrombolysis.

**Variable**	**All patients**	**Tirofiban**	**No-tirofiban**	***P*-value**	**Odds ratio**
	**(*n =* 73)**	**(*n =* 59)**	**(*n =* 14)**		**(95% CI)**
mRS (≤ 2) at 7 days	21 (28.77)	21 (35.59)	0	0.008	1.37 (1.16–1.61)
mRS (≤ 2) at 90 days	37 (50.68)	37 (62.71)	0	< 0.001	1.64 (1.26–2.12)

Data is shown in n (%). mRS, modified Rankin Scale, CI, confidence interval.

**Figure 3:**
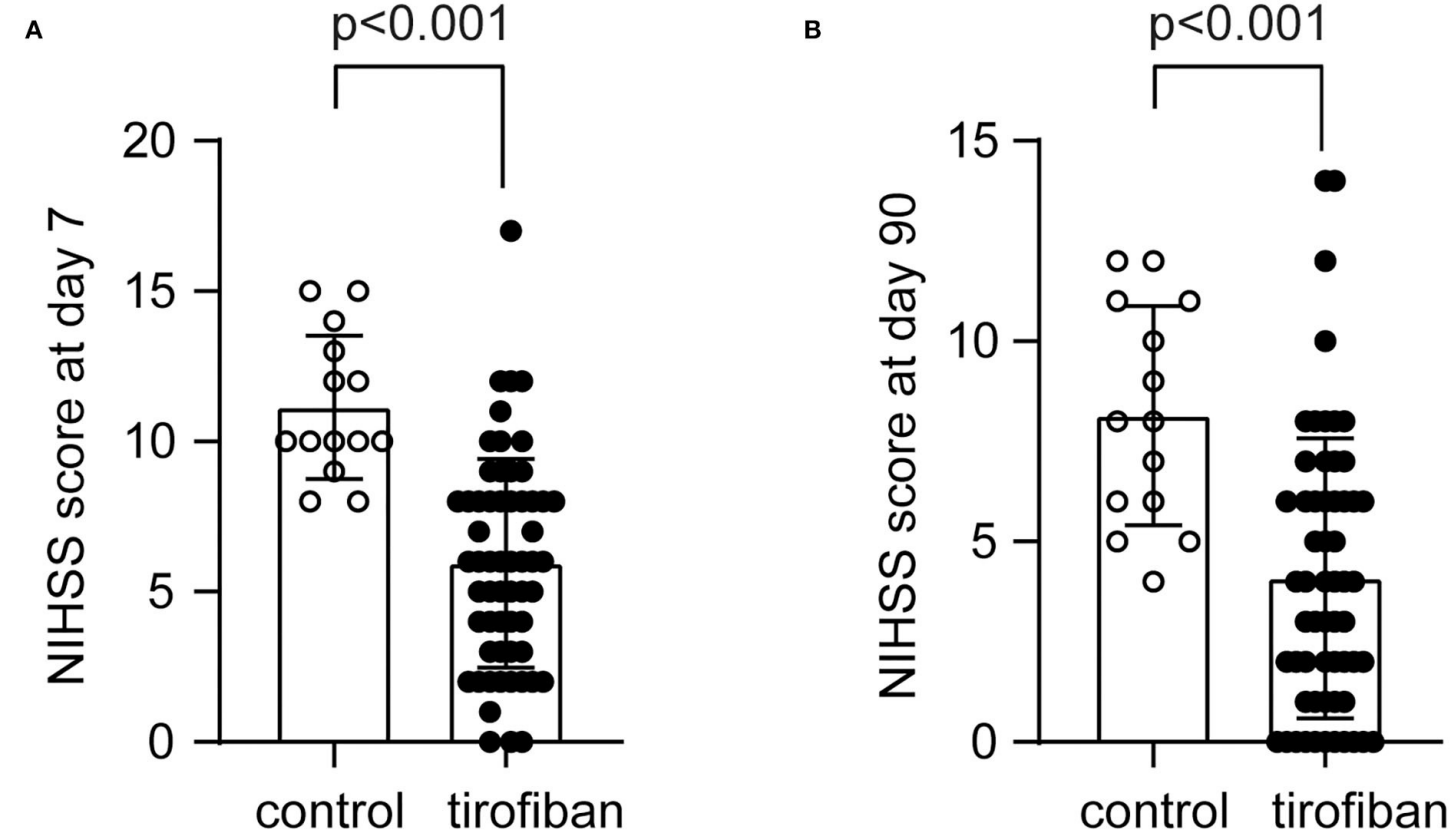
Tirofiban treatment reduces National Institutes of Health stroke scale (NIHSS) scores at 7 and 90 days. The neurological deficits of AIS patients were assessed with NIHSS at both 7 and 90 days. The NIHSS scores range from 0 to 42, indicating from no stroke symptoms to severe stroke. Tirofiban treatment reduces NIHSS scores at both day 7 **(A)** and 90 **(B)** compared with the control groups without tirofiban treatment. Two-independent group Mann-Whitney *U* test*, n* = 59 and 14 for tirofiban and control groups, respectively.

Similarly, we evaluated the severity of neurological deficits of END patients treated with and without tirofiban by NIHSS score at days 7 and 90. Compared with regular treatment, additional treatment with tirofiban within 24 h after intravenous thrombolysis reduced NIHSS scores from 11.14 ± 2.38 to 5.95 ± 3.48 as early as day 7 (Figure 3A; *U* = 85,500, *Z* = −4.612, *P* < 0.001). At day 90, NIHSS scores in the control group (8.14 ± 2.74) were also significantly higher than those (4.08 ± 3.50) in the tirofiban-treated group (Figure 3B; *U* = 148,500, *Z* = - 3.725, *P* < 0.001).

### Safety outcome

To evaluate the safety of tirofiban treatment, we recorded all adverse events for END patients treated with and without tirofiban, which occurred during the 90-day follow-up period, both in the hospital and at home. None of the patients died before the end of the study. As shown in Table 3, we found no differences between the tirofiban and control groups in the incidence of symptomatic or non-symptomatic intracerebral hemorrhage detected by CT or MRI scans of the head, or in the incidence of extracranial hemorrhage (e.g., gastrointestinal hemorrhage, urethrorrhage, ecchymoma, or oral or nasal mucosal bleeding) within 7 days after intravenous thrombolysis. In addition, treatment with tirofiban neither decreased platelet count in the blood nor induced pneumonia, and other non-hemorrhagic serious adverse events (e.g., cerebral herniation, respiratory and circulatory disorders, urinary tract infections, sepsis, hepatic and renal failure, acute coronary syndrome, venous thrombosis, psychiatric symptoms).

**Table 3:** Safety of tirofiban use in END patients after intravenous thrombolysis.

**Variable**	**All patients**	**Tirofiban**	**No-tirofiban**	***P*-value**	**Odds ratio**
	**(*n =* 73)**	**(*n =* 59)**	**(*n =* 14)**		**(95% CI)**
Symptomatic intracerebral hemorrhage	2 (2.74)	2 (3.39)	0	0.485	0.80 (0.72–0.90)
Asymptomatic intracerebral hemorrhage	4 (5.48)	3 (5.08)	1 (7.14)	0.761	0.70 (0.07–7.25)
Extracranial bleeding	6 (8.22)	5 (8.247)	1 (7.14)	0.870	1.20 (0.13–11.20)
Thrombocytopenia	2 (2.74)	2 (3.39)	0	0.485	0.80 (0.72–0.90)
Pneumonia	17 (23.29)	16 (27.12)	1 (7.14)	0.112	4.84 (0.58–40.03)
Other non-hemorrhagic severe adverse events	16 (21.92)	15 (25.42)	1 (7.14)	0.137	4.43 (0.53–36.80)
Deaths within 90 days	0	0	0	–	–

Data is shown in n (%). CI, confidence interval.

### Multivariate analysis of the impact of tirofiban treatment on the outcome of AIS patients with END

As shown in Table 1, some variables (e.g., the proportion of patients with coronary artery disease or smoking, serum homocysteine level, and TOAST classification) tended to differ (*P* < 0.10) between the tirofiban and control groups. To further investigate the therapeutic efficacy of tirofiban in AIS patients with END and to exclude effects from potential cofounding factors, we performed an ordinal logistic regression analysis with mRS at day 90 (disability scale 0–5) as the ordinal dependent variable. A total of 4 models were constructed using tirofiban treatment, in combination with pre-stroke vascular risk factors (model 1), clinical (model 2) and laboratory (model 3) findings before initiation of tirofiban treatment, and all adverse events (model 4) observed during the 90-day follow-up period, as independent variables. As shown in Table 4, treatment with tirofiban (1 = treatment and 0 = non-treatment) correlated negatively with mRS scores (from 0 [no symptoms] to 5 [severe disability]) at day 90 (*P* ≤ 0.001) in all 4 models, indicating that treatment with tirofiban decreased mRS scores and improved functional recovery. It was not surprising that the higher NIHSS score at admission (model 2) and, in particular, the occurrence of symptomatic intracerebral hemorrhage (model 4) independently predicted higher mRS scores (poor recovery) in AIS patients (*P* < 0.05). Interestingly, higher blood HDL and LDL levels at admission were correlated with lower mRS scores (*P* < 0.05). There are studies showing an association between high blood LDL cholesterol levels and favorable functional outcomes in AIS patients after reperfusion therapy (17, 18), although the actual relationship between serum LDL and the clinical outcome in AIS patients needs further investigation.

**Table 4:** Multivariate regression analysis between tirofiban and mRS at day 90.

**Independent variable**	**Estimate (95% CI)**	***P*-value**
**Model 1 (Prestroke vascular risk factors)**		
Tirofiban treatment	−2.17 (−3.45 to −0.89)	0.001
Age, year	0.03 (−0.01 to 0.07)	0.128
Women	−0.39 (−1.84 to 1.07)	0.602
Hypertension	−0.17 (−1.20 to 0.85)	0.739
Diabetes mellitus	−0.50 (−1.67 to 0.67)	0.403
Dyslipidemia	0.62 (−0.43 to 1.66)	0.246
Previous stroke	0.36 (−0.89 to 1.60)	0.577
Coronary artery disease	−0.44 (−1.54 to 0.67)	0.441
Current smoking	−0.45 (−1.72 to 0.83)	0.491
**Model 2 (Clinical findings at admission)**		
Tirofiban treatment	−2.55 (−3.83 to −1.27)	< 0.001
SBP at baseline, mmHg	0.20 (−0.01 to 0.05)	0.142
DBP at baseline, mmHg	0.002 (−0.03 to 0.03)	0.908
Pre-stroke antiplatelet use	0.003 (−1.03 to 1.02)	0.995
Admission NIHSS score	0.20 (0.03 to 0.37)	0.020
Increase of NIHSS score from admission after IVT	0.05 (−0.31 to 0.41)	0.778
Stroke onset to IVT, minutes	−0.001 (−0.01 to 0.01)	0.830
Large artery atherosclerosis (TOAST classification)	−0.05 (−0.95 to 0.85)	0.915
**Model 3 (Laboratory findings at admission)**		
Tirofiban treatment	−2.66 (−3.90 to −1.42)	< 0.001
Glucose, mg/dL	0.07 (−0.14 to 0.28)	0.505
TC, mmol/L	0.28 (−0.13 to 0.68)	0.182
TG, mmol/L	0.17 (−0.42 to 0.75)	0.581
LDL, mmol/L	−0.64 (−1.21 to −0.07)	0.029
HDL, mmol/L	−2.03 (−3.47 to −0.58)	0.006
Homocysteine, μmol/L	0.03 (−0.01 to 0.07)	0.140
WBC, × 10^9^/L	−0.08 (−0.28 to 0.11)	0.410
Platelet, × 10^9^/L	0.001 (−0.01 to 0.01)	0.901
Fibrinogen, g/L	0.12 (−0.42 to 0.65)	0.669
**Model 4 (Complications)**		
Tirofiban treatment	−2.85 (−4.14 to −1.56)	< 0.001
Symptomatic intracerebral hemorrhage	18.727 (14.02 to 23.44)	< 0.001
Asymptomatic intracerebral hemorrhage	−0.26 (−2.41 to 2.94)	0.847
Extracranial bleeding	0.51 (−2.39 to 1.38)	0.598
Thrombocytopenia	−19.21 (−19.21 to −19.21)	-
Pneumonia	0.87 (14.02 to 23.44)	0.440
Other non-hemorrhagic severe adverse events	1.11 (−1.17 to 3.38)	0.598

DBP indicates diastolic blood pressure; HDL, high-density lipoprotein; IVT, intravenous thrombolysis; LDL, low-density lipoprotein; NIHSS, National Institutes of Health Stroke Scale; SBP, systolic blood pressure; TC, total cholesterol; TG, triglyceride; TOAST, Trial of ORG 10172 in Acute Stroke Treatment; and WBC, white blood cell.

## Discussion

Ischemic stroke is a leading cause of disability and death in the elderly. Improvement of reperfusion therapy in AIS patients, especially prevention of END after intravenous thrombolysis, is desirable. In this prospective pilot study, we treated AIS patients with tirofiban for 24 h immediately after the diagnosis of END, and observed that tirofiban together with subsequent regular antiplatelet therapy (e.g., aspirin and/or clopidogrel) significantly attenuated neurological deficits and improved functional recovery at both 7 and 90 days.

The main cause of ischemic stroke is atherosclerosis that develops from accumulated lipids and lipid-laden macrophages in the arterial wall. When atherosclerotic plaques rupture, the exposed thrombogenic materials lead to platelet activation, aggregation, thrombosis and vessel occlusion (19). It has been observed that platelet activation, as evidenced by increased mean platelet volume, begins in AIS patients (20). The increase in platelet volume also predicts poor recovery of AIS patients at 3 months (21, 22). Thus, inhibition of platelet activation has become the standard therapeutic strategy for AIS patients.

It has been hypothesized that END in AIS patients after intravenous thrombolysis may result from the extension of the initial thrombus (5). In the imaging of susceptibility vessel sign with T2^*^ magnetic resonance, thrombi spread more frequently within 24 h after intravenous thrombolysis in AIS patients with END than in AIS patients without END (23). Consistent with the thrombosis hypothesis, taking aspirin and clopidogrel before stroke onset potentially protects against unexplained END and improves the functional recovery of AIS patients (24–26). Therefore, antithrombotic drugs such as tirofiban may prevent END. We and other groups (8, 27) did observe that treatment with tirofiban within 24 h of intravenous thrombolysis improves the functional outcome of AIS patients with END in 3 months.

It has been observed that tirofiban infusion is more beneficial for AIS patients with large artery atherosclerosis than for patients with small vessel occlusion (16), which may be due to the different degree of platelet activation in these two groups of patients. As discussed above, rupture of atherosclerotic plaques in large arteries is the main activator of platelets (19). Platelets are also activated by increased shear stress at the site of stenosis lesion in cerebral arteries (28–30). Our study showed an OR of 1.92 for treatment with tirofiban to improve functional recovery at day 90 in END patients with large artery atherosclerosis alone, which was higher than the OR (1.64) of tirofiban treatment in END patients with occlusion in large or small vessels. This is consistent with the previous observation (16). Because the percentage of END patients in the tirofiban treatment group tended to be smaller than in the control group (*P* = 0.080; see Table 1), our study may underestimate the therapeutic efficacy of tirofiban treatment in END patients.

Compared with aspirin, which irreversibly inhibits cyclooxygenase-1 (COX1), thereby blocking thromboxane A2 production and the subsequent hemostatic cascade, tirofiban specifically inhibits the cross-linking of glycoprotein IIb/IIIa on adjacent platelets by fibrinogen and therefore may have less adverse effects on normal hemostasis (31). Abciximab, a chimeric monoclonal antibody targeting glycoprotein IIa/IIIb receptor, has also been used to treat AIS patients; unfortunately, abciximab significantly increased the risk of intracranial hemorrhage (32). It should be noted that abciximab maintains the anti-platelet effect for 72–96 h, much longer than tirofiban (4 h) after drug discontinuation (33). In our study, we did not observe an increase in hemorrhagic events inside and outside the brain in tirofiban-treated AIS patients, confirming the findings of other groups (8, 9, 27). Since tirofiban alone or together with rt-PA was used to prevent the progression of ischemic lesions and microembolism in AIS patients 20 years ago (34–37), there is sufficient evidence that treatment with tirofiban does not increase the risk of intracerebral hemorrhage (38). Tirofiban has been increasingly used in China in combination with both intravenous thrombolysis and intra-arterial thrombectomy. All these studies have shown that treatment with tirofiban promotes the cerebral artery recanalization and functional recovery in AIS patients without an increase in hemorrhagic complications (39–44). Therefore, tirofiban has been recommended in the Chinese Stroke Association guidelines for the use in bridging intravenous thrombolysis and thrombectomy, or in the perioperative phase of endovascular therapy (13). However, it is important to note that treatment with tirofiban may not benefit cardioembolic stroke patients (40). Intra-arterial infusion of tirofiban potentially increases intracerebral hemorrhage (41). There was also a study showing that continuous intravenous administration of tirofiban in addition to endovascular therapy did not improve recanalization but increased intracerebral hemorrhage and lad to poor recovery in AIS patients (45).

The small size of the studied population is, of course, the major limitation of our study, which may lead to the observation that: (1) no patients receiving regular antiplatelet therapy achieved favorable functional recovery (mRS ≤ 2); and (2) no patients receiving regular therapy developed intracerebral hemorrhage. However, in statistical analysis of continuous variables, there is a “rule of 12” that states that increasing the sample size to 12 participants significantly improves the precision of the mean and variance of a research population, whereas increasing the sample size beyond 12 participants does not (10). Therefore, this size of 12 participants is feasible for most early stage researchers to conduct the study in a single center and still obtain valuable preliminary information. In addition, there have been too few clinical trials of tirofiban in AIS patients outside China. The lack of comparisons between results from different regions makes it difficult to assess the true therapeutic efficacy of tirofiban.

## Conclusion

Our prospective, randomized, and open-label pilot study suggests that treatment with tirofiban within 24 h of intravenous rt-PA thrombolysis has the potential to rapidly reduce neurological deficits and improve functional outcome without increasing the risk of various hemorrhagic complications in AIS patients who present with neurological deterioration after recanalization therapy. However, our pilot study shows nothing about the actual therapeutic efficacy of tirofiban infusion in AIS patients to prevent stroke progression after intravenous thrombolysis, but demonstrates the feasibility and acceptability of a prospective, multicenter clinical trial of tirofiban with a large population of AIS patients.

## Data availability statement

The original contributions presented in the study are included in the article/supplementary material, further inquiries can be directed to the corresponding author/s.

## Ethics statement

The studies involving human participants were reviewed and approved by the Kunshan Affiliated Hospital of Jiangsu University. The patients/participants provided their written informed consent to participate in this study.

## Author contributions

YZ designed the study. YZ, JW, ZM, GM, DL, YLi, XQ, LuZ, FS, LeZ, and JY collected the main study data. YLiu analyzed the main study data and wrote the paper. All authors contributed to the article and approved the submitted version.

## Funding

This work was supported by the Suzhou development plan for science and technology in 2020 (SYSD2020033, to YZ) Key R&D program in Kunshan City (KS2206, to YZ), and New technique program from the first people's hospital of Kunshan (2020, Class I, No. 01).

## Conflict of interest

The authors declare that the research was conducted in the absence of any commercial or financial relationships that could be construed as a potential conflict of interest.

## Publisher's note

All claims expressed in this article are solely those of the authors and do not necessarily represent those of their affiliated organizations, or those of the publisher, the editors and the reviewers. Any product that may be evaluated in this article, or claim that may be made by its manufacturer, is not guaranteed or endorsed by the publisher.
